# The effectiveness of desogestrel for endometrial protection in women with abnormal uterine bleeding-ovulatory dysfunction: a non-inferiority randomized controlled trial

**DOI:** 10.1038/s41598-022-05578-0

**Published:** 2022-01-31

**Authors:** Nisarath Soontrapa, Manee Rattanachaiyanont, Malee Warnnissorn, Thanyarat Wongwananuruk, Suchada Indhavivadhana, Prasong Tanmahasamut, Kitirat Techatraisak, Surasak Angsuwathana

**Affiliations:** 1 Gynecologic Endocrinology Unit, Department of Obstetrics and Gynecology, Faculty of Medicine Siriraj Hospital, Mahidol University, Bangkok, 10700 Thailand; 2 Department of Pathology, Faculty of Medicine Siriraj Hospital, Mahidol University, Bangkok, Thailand

**Keywords:** Randomized controlled trials, Endocrine reproductive disorders

## Abstract

Women with chronic abnormal uterine bleeding-ovulatory dysfunction (AUB-O) are at increased risk of endometrial neoplasia. We conducted a non-inferiority randomized controlled trial to determine the effectiveness of two cyclic-progestin regimens orally administered 10 d/month for 6 months on endometrial protection and menstruation normalization in women with AUB-O. There were 104 premenopausal women with AUB-O randomized to desogestrel (DSG 150 µg/d, n = 50) or medroxyprogesterone acetate (MPA 10 mg/d, n = 54) group. Both groups were comparable in age (44.8 ± 5.7 vs. 42.5 ± 7.1 years), body mass index (24.8 ± 4.7 vs. 24.9 ± 4.7 kg/m^2^), and AUB characteristics (100% irregular periods). The primary outcome was endometrial response rate (the proportion of patients having complete pseudodecidualization in endometrial biopsies during treatment cycle-1). The secondary outcome was clinical response rate (the proportion of progestin withdrawal bleeding episodes with acceptable bleeding characteristics during treatment cycle-2 to cycle-6). DSG was not inferior to MPA regarding the endometrial protection (endometrial response rate of 78.0% vs. 70.4%, 95% CI of difference − 9.1–24.4%, non-inferiority limit of − 10%), but it was less effective regarding the menstruation normalization (acceptable bleeding rate of 90.0% vs 96.6%, *P* = 0.016).

**Clinical trial registration**: ClinicalTrials.gov (NCT02103764, date of approval 18 Feb 2014).

## Introduction

Abnormal uterine bleeding (AUB) associated with anovulation without gross uterine abnormality is classified as “abnormal uterine bleeding-ovulatory dysfunction” or AUB-O in the International Federation of Gynecology and Obstetrics (FIGO) classification^1^. AUB-O is a common gynecologic complaint in women during perimenarcheal or perimenopausal periods, and those with polycystic ovary syndrome or obesity^2–5^. Women with AUB-O usually have irregular menstrual periods with or without heavy bleeding. The cases with chronic AUB-O are at increased risk of endometrial neoplasia because of prolonged exposure to unopposed estrogen. Therefore, the treatment of AUB-O aims to normalize menstruation and protect the endometrium from endometrial neoplasia^2^. Despite the paucity of evidence from randomized controlled trials (RCT), the current treatment options include estrogen-progestin and progestin-only therapy^6^. The former, in the form of combined hormonal contraceptives, is best for sexually-active women with a need for contraception^7^. The latter is preferable for women who are in the perimenopausal period, or who are intolerant to or contraindicated to combined hormonal contraceptives.

The commonly used progestin for AUB-O is medroxyprogesterone acetate (MPA, 5–10 mg/d, 10–14 d/month)^1^. Its strong progestogenic effect is preferable for endometrial protection, but its undesirable glucocorticoid, mineralocorticoid, and androgenic effects may negatively affect glucose and lipid metabolism in long-term users^8–11^. A report from a clinical study in 1990 suggested that cyclic progestin (i.e., MPA, norethindrone, or norethisterone), given for 12–14 days each month, was effective management for most women with anovulatory dysfunctional uterine bleeding^12^. Nowadays, there are a variety of progestins that may be useful for the treatment of AUB-O. Each progestin has its own unique properties, benefits, and disadvantages^11^. Therefore, prior to adopting a new drug as a treatment option, its efficacy should be proven by an RCT.

Desogestrel (DSG), a third-generation progestin, has high progestogenic activity with low androgenic and little to no glucocorticoid and mineralocorticoid effects^13,14^. DSG may, therefore, be a better choice for perimenopausal or obese women who are at increased risk for abnormal glucose and lipid metabolism. Information specific to DSG therapy for AUB-O is lacking. This study aimed to evaluate the effectiveness of cyclic DSG compared with cyclic MPA on endometrial protection and menstruation normalization in women with AUB-O. We also assessed the safety of these two progestins on metabolic parameters after 6 months of treatment.

## Results

### Participants

**Figure 1:**
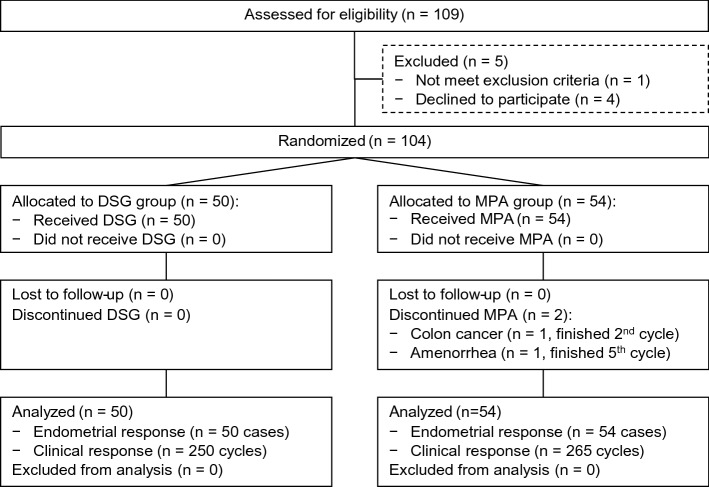
CONSORT participant flow. *DSG* desogestrel, *MPA* medroxyprogesterone acetate.

Figure 1 shows flow of study participants. Of 109 eligible patients, 104 cases were enrolled and randomized to the study group (DSG, n = 50) or the control group (MPA, n = 54). According to the exclusion criteria, one case was excluded, and four cases declined to participate. There were two dropouts in the MPA group; one case after treatment cycle-2 because of colon cancer, and the other after cycle-5 because of amenorrhea. Their missing data regarding withdrawal bleeding were imputed from the last observation carried forward. Therefore, there were five imputed data, including four cycles of normal withdrawal bleeding and one cycle of no withdrawal bleeding; the data of which contributed to the clinical outcome of the MPA group.

Table 1 shows demographic and baseline characteristics of the 104 intention-to-treat participants. The DSG and MPA groups were comparable in age (44.8 ± 5.7 vs. 42.5 ± 7.1 years), body mass index (BMI, 24.8 ± 4.7 vs. 24.9 ± 4.7 kg/m^2^), education > 12 years (64.0% vs. 61.1%), and AUB characteristics (irregular bleeding of 100%). Both groups were comparable in baseline metabolic parameters, including fasting blood glucose, total cholesterol, triglycerides, high-density lipoprotein cholesterol (HDL-C), and low-density lipoprotein cholesterol (LDL-C) levels. The non-compliance rate was low in both groups (2.8% in the DSG and 2.6% in the MPA groups).

**Table 1:** Demographic and baseline characteristics of 104 intention-to-treat patients.

Characteristics	DSG (N = 50)	MPA (N = 54)
Age (years)	44.8 ± 5.7	42.5 ± 7.1
Body mass index (kg/m^2^)	24.8 ± 4.7	24.9 ± 4.7
Waist circumference (cm)	80.6 ± 9.5	79.6 ± 9.9
Parity, median (range)	2 (0 to 4)	1 (0 to 4)
Systolic BP (mmHg)	121.7 ± 16.4	124.9 ± 17.0
Diastolic BP (mmHg)	72.4 ± 10.7	72.6 ± 10.9
Education > 12 years	32 (64.0)	33 (61.1)
Medical diseases (at least one)	11 (22.0)	11 (20.4)
Autoimmune diseases	2 (4.0)	1 (1.9)
Hypertension	4 (8.0)	4 (7.4)
Thyroid diseases	5 (10.0)	3 (5.6)
Others	0 (0)	3 (5.6)
Character of abnormal uterine bleeding		
Irregular bleeding	36 (72.0)	47 (87.0)
Irregular and excessive bleeding	14 (28.0)	7 (13.0)
Metabolic parameters		
Fasting blood glucose (mg/dL)	89 (83 to 93)	85 (80 to 90)
Total cholesterol (mg/dL)	198 (178 to 220)	196 (171 to 216)
Triglycerides (mg/dL)	81 (60 to 106)	91 (67 to 143)
HDL-C (mg/dL)	63 (48 to 73)	65 (54 to 77)
LDL-C (mg/dL)	116 (103 to 137)	103 (86 to 129)

	(N = 250)	(N = 265)
Non-compliance treatment cycles		
Skipped doses > 20%	7 (2.8)	7 (2.6)

Data are mean ± standard deviation, median and interquartile range (in parentheses), or number and percentage (in parentheses), otherwise specified.

*BP* blood pressure, *DSG* desogestrel, *HDL-C* high-density lipoprotein cholesterol, *LDL-C* low-density lipoprotein cholesterol; MPA, medroxyprogesterone acetate.

### Efficacy outcomes

**Figure 2:**
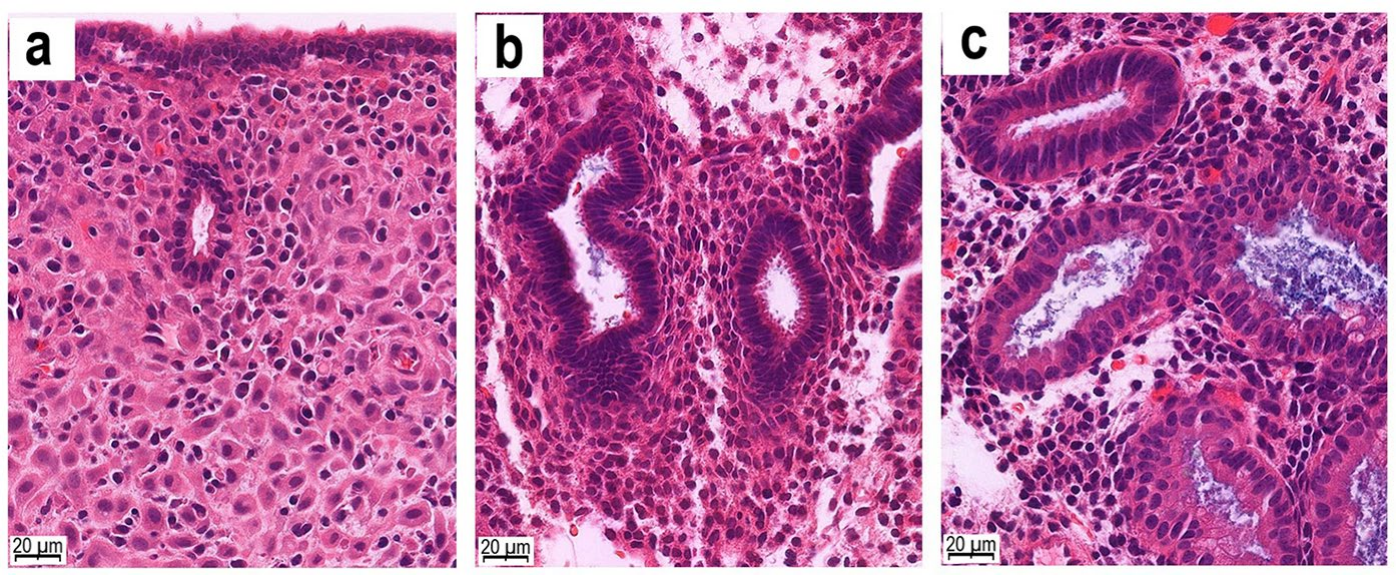
Histopathology of endometrial response after treatment. (**a**) Complete transformation showing a small inactive gland with pseudodecidual stromal change, (**b**) Proliferative endometrium without response, (**c**) Inadequate secretory change and inconspicuous pseudodecidual stromal change.

Figure 2 and Table 2 show efficacy outcomes. Figure 2 shows various degrees of endometrial response to treatment. The treatment is considered effective if the post-treatment endometrium shows a small inactive gland with pseudodecidual stromal change, as in Fig. 2a. The endometrial response to DSG seemed to be slightly better than that to MPA as determined by the proportion of complete pseudodecidualization (78.0% *vs.* 70.4%, *P* = 0.375). The 95% CI of difference (Δ) of this outcome (− 9.1%, 24.4%) indicated the non-inferiority of DSG to MPA as its lower bound lay above the non-inferiority limit of − 10%.

**Table 2:** Efficacy outcomes.

Outcomes	DSG		MPA		*P*
	N	n (%)	N	n (%)	
Endometrial response (cases)	50		54		0.375
Complete pseudodecidualization		39 (78.0)		38 (70.4)	
Partial or no pseudodecidualization		11 (22.0)		16 (29.6)	
Clinical response (cycles)	250		265		0.016
Acceptable bleeding		225 (90.0)		256 (96.6)	
Prolonged bleeding		25 (10.0)		9 (3.4)	

The endometrial response rates were compared using chi-square test; whereas the clinical response rates were compared using generalized estimating equations (GEE). A *P*-value < 0.05 indicates statistical significance.

For endometrial response, 95% CI of difference (Δ) in the proportion of complete pseudodecidualization is (− 9.1% to 24.4%); the lower bound of which lies above the non-inferiority limit of − 10%

For clinical response, acceptable bleeding includes treatment cycles with normal withdrawal bleeding (amount and duration) and treatment cycles without withdrawal bleeding.

*DSG* desogestrel, *MPA* medroxyprogesterone acetate.

Both DSG and MPA effectively normalized bleeding to an acceptable level (normal or no withdrawal bleeding) for > 90% of cases. However, the clinical response to DSG was not as good as that to MPA, as the proportion of acceptable bleeding was significantly lower in the DSG group than in the MPA group (90.0% vs. 96.6%, *P* = 0.016). In other words, the DSG group had a significantly higher rate of prolonged bleeding (10.0% vs. 3.4%).

### Safety and tolerability

Table 3 shows safety and tolerability data which presents the incidence of adverse events and side effects, and the changes from baseline to month-6 of treatment (δ) for various metabolic parameters. One patient in the MPA group was found to have colon cancer after treatment cycle-2 and was withdrawn from the trial. The most common side effect was drowsiness, which approximately one-third of patients reported in both groups. The incidence of nausea was clinically significantly higher in the DSG group (12.0% vs*.* 1.9%, *P* = 0.053). Both DSG and MPA groups were comparable in the δ of every metabolic parameter. Most of the δ had a minus sign indicating favorable changes for total cholesterol, triglycerides, and LDL-C, but an unfavorable change for HDL-C. None of the metabolic parameters had a δ beyond ± 10%; therefore, the δ was not clinically important.

**Table 3:** Safety and tolerability.

	N	DSG	N	MPA	*P*
Serious adverse events	50	0 (0)	54	1 (1.9)	1.000
Side effect (at least one)	50	29 (58.0)	54	29 (53.7)	0.659
Breast pain	50	6 (12.0)	54	3 (5.6)	0.307
Dermatologic problems	50	11 (22.0)	54	10 (18.5)	0.659
Drowsiness	50	14 (28)	54	13 (24)	0.648
Fever-like symptom	50	0 (0)	54	1 (1.9)	1.000
Increased appetite	50	5 (10.0)	54	9 (16.7)	0.320
Nausea	50	6 (12.0)	54	1 (1.9)	0.053
Pelvic cramp	50	0 (0)	54	2 (3.7)	0.496
Rash	50	1 (2.0)	54	2 (3.7)	1.000
δ of metabolic parameters					
Fasting blood glucose (mg/dL)	50	0 (− 4.3 to 4.3)	52	0.5 (− 2.8 to 4.8)	0.840
Total cholesterol (mg/dL)	50	− 8.5 (− 25.5 to 3.3)	52	− 0.5 (− 12.0 to 12.0)	0.072
Triglycerides (mg/dL)	50	− 7.5 (− 21.3 to 9.5)	52	− 6 (− 24.0 to 14.5)	0.723
HDL-C (mg/dL)	50	− 5 (− 10.3 to 0.3)	52	− 3.5 (− 12.0 to 1.8)	0.776
LDL-C (mg/dL)	50	− 1.7 (− 20.6 to 8.4)	51	5.6 (− 10 to 18.4)	0.159

The incidence of serious adverse events and side effects are presented in number and percentage (in parentheses). The changes from baseline to month-6 of treatment (δ) of metabolic parameters are presented in median and interquartile range (in parentheses). Categorical data were compared using chi-square test or Fisher’s exact test. Continuous data were compared using Mann–Whitney U test.

The serious adverse event included one case of colon cancer in the MPA group. Dermatologic problems included acne, pigmentation, or oily skin.

*DSG* desogestrel, *MPA* medroxyprogesterone acetate, *HDL-C* high-density lipoprotein cholesterol, *LDL-C* low-density lipoprotein cholesterol.

## Discussion

Treatment for AUB-O aims to restore normal physiological change of the endometrium to prevent endometrial neoplasia and normalize menstruation^2^. There is a lack of evidence from randomized controlled trials, especially about the effect of treatment for AUB-O on endometrial histopathology. Most studies showed only intermediate outcomes, such as menstrual cycle regularization^15–17^. Our present study showed that two cyclic-progestin regimens (DSG 150 µg/d or MPA 10 mg/d, 10 d/month) could transform endometrium to complete pseudodecidualization in > 70% of cases in the treatment cycle-1 and could normalize menstruation in > 90% of all treatment cycles.

Obese women with AUB-O are at high risk for developing endometrial neoplasia and may benefit from preventive measures^18^. Although authorities recommend that endometrial biopsy should be performed in women older than 45 years^19,20^, obese women may need endometrial biopsy at a younger age^18^. Our study population was slightly younger than 45 years, but they had BMI in the overweight to obese classification according to the WHO Asia Pacific BMI cut point (≥ 23 kg/M^2^) ^21^. The preventive measures for these women include bodyweight reduction by lifestyle modification and progestin therapy^18^.

Progestin therapy is available in various forms, including combined hormonal contraceptives, progestin-only contraceptives, and cyclic-progestin therapy. Each form has its own benefits and drawbacks. The progestin therapy results in pseudodecidualization of the endometrium. A case–control study reported that progestins in most combined oral contraceptives were adequate to protect against endometrial cancer^22^. DSG in the form of progestin-only oral contraceptive might also have this protective effect as it contains a similar dosage of DSG as a combined oral contraceptive does. It is unknown whether cyclic progestin therapy in premenopausal women with AUB-O also retains this protective effect. Evidence from menopausal hormone therapy shows that continuous combined estrogen-progestin regimens reduce the risk of endometrial cancer while cyclic regimens do not alter the risk^23^. We presume that the risk of endometrial cancer in women with AUB-O treated with cyclic progestin may be the same as the risk in women with regular ovulation.

The equivalent dosages of progestins that are adequate for endometrial transformation were reviewed by Schindler, et al.^11^ The authors summarized the daily and total doses of progestins. They considered MPA 5–10 mg/d for a total dose of 80 mg and DSG 150 µg/d for a total dose of 2 mg to be equivalent. In the present study, a total dose of 100 mg MPA or 1.5 mg DSG was used within 10 days. Although the total dose of 1.5 mg DSG was less than that suggested by Schindler’s review, this dosage was as effective as 100 mg MPA. The 10-day application of one or the other progestins could not completely transform the endometrium in some patients. Ineffective endometrial transformation may be due to the inadequate cumulative effect of progestin: dosage and duration^2^. It is suggested that the duration of cyclic progestin therapy should be 12–14 days per cycle to protect the endometrium effectively^2,12^. However, this recommendation was based on information from the first-generation progestins. Therapy with more potent third-generation progestins may protect the endometrium with a shorter duration of treatment. We found that 10-day DSG seemed to be more effective than 10-day MPA (78% vs. 70% complete pseudodecidualization). However, our study did not have enough power to show the superiority of DSG over MPA in this regard.

Despite the observed comparable effectiveness relating to endometrial response, MPA was superior to DSG for clinical response. The drawback of DSG was the higher rate of bleeding for longer than eight days (11.1% vs*.* 4.6%). Some participants might have concomitant structural lesions as a confounding cause for prolonged withdrawal bleeding. In the present study, transvaginal ultrasonography could exclude gross structural lesions but not a tiny endometrial polyp which might be detected by saline infusion sonohysterography or hysteroscopy. With the strength of RCT, this confounder should be distributed equally between the groups. Interestingly, the finding that MPA had better bleeding control than DSG was consistent with our previous study that found MPA superior to other progestins regarding menstruation normalization in women with non-atypical endometrial hyperplasia^24^. Since each progestin has its own unique profiles^10,25,26^, each may act differently on molecular microenvironment involving hemostasis of endometrium, which may cause different uterine bleeding patterns.

Some progestins have an adverse effect on metabolic profiles, although most of the evidence is derived from studies of menopausal hormone therapy. A review in 2017 regarding progestins and blood lipids found that MPA mitigated the beneficial effects of menopausal hormone therapy on blood lipids^27^. In our study, we expected the metabolic profile to be better in the DSG group than in the MPA group because DSG has a low androgenic effect and little to no glucocorticoid or mineralocorticoid effect. However, the change in all metabolic parameters from baseline to month-6 was marginal and may not be clinically meaningful. It was possible that cyclical application of these two progestins for 10 d/month did not have an adverse effect on metabolic parameters.

The strength of the present study is its prospective randomized controlled design that can prevent bias. Moreover, we evaluated both endometrial and clinical responses to ensure that the treatment not only normalized menstruation but also protected the endometrium. However, RCT has a limitation in generalizability. We evaluated two cyclic-progestin regimens in overweight premenopausal women with AUB-O and followed up them for 6 months. Application of these regimens to patients with different characteristics in a longer duration of treatment may result in different efficacy and safety outcomes.

In conclusion, according to our definition of non-inferiority, the cyclical administration of DSG (150 µg/d, 10 d/month) is not inferior to MPA (10 mg/d, 10 d/month) for pseudodecidualization of endometrium in women with AUB-O. Both DSG and MPA are effective for menstruation normalization in > 90% of cases, but DSG is less effective than MPA. The 6-month metabolic profile changes are comparable between the DSG and MPA groups, and are not clinically meaningful. Therefore, cyclic DSG can be used as an alternative to MPA for endometrial protection and menstruation normalization in AUB-O.

## Methods

This non-inferiority double-blind RCT was conducted at the Gynecologic Endocrinology Unit, Department of Obstetrics and Gynecology, Faculty of Medicine Siriraj Hospital, Mahidol University, Bangkok, Thailand, from 11 March 2014 to 30 November 2018. This trial was a part of the Siriraj Abnormal Uterine Bleeding (SI-AUB) project, an ongoing project established in 2010. The SI-AUB project comprises many sub-studies, including observational studies and clinical trials. The SI-AUB project and this study were conducted in accordance with the principles set forth in the Declaration of Helsinki.

### Participants

Participants were premenopausal women who were newly diagnosed with AUB-O. A diagnosis of AUB-O was made if a patient had unremarkable findings from pelvic ultrasonogram and endometrial histopathology revealed no structural lesions^28^. Regarding the endometrial histopathology, only patients with proliferative endometrium were recruited. A participant was excluded if she had conditions that might cause AUB (e.g., having coagulation defect or taking certain medications), took hormonal medication within 3 months before enrollment, planned to get pregnant within 6 months, needed hormonal contraception, had contraindications for progestogen administration (e.g., history of breast cancer, severe liver disease, or progestogen allergy), or had diabetes mellitus and/or dyslipidemia with ongoing treatment. All sexually active participants were advised to use barrier contraception. An enrolled participant would be withdrawn from the study if she had a follow-up endometrial histopathology report of endometrial neoplasia or she encountered a serious adverse event. This participant would be counted as a case of treatment failure in the intention-to-treat analysis.

### Study procedures

Eligible participants were recruited from the gynecology outpatient department. Routine evaluation for AUB at our clinic includes clinical evaluation and pelvic ultrasonography. Endometrial histopathology is indicated for women at increased risk of endometrial neoplasia, e.g., older than 45 years, irregular menstruation in women with polycystic ovary syndrome, or obesity. Demographic characteristics, medical history, menstrual bleeding pattern, concomitant medications, and findings of general and gynecologic examinations were collected using a structured record form. Transvaginal ultrasonography was performed to assess the structure of pelvic organs. When the ultrasound probe could not be inserted vaginally, transrectal ultrasonography would be performed instead. Endometrial tissue was obtained by an office endometrial biopsy procedure using a flexible instrument. Patients were then scheduled two weeks after the procedure to be enrolled into the present study or another sub-study of the SI-AUB project.

The enrolled participants were scheduled for five visits, including ***visit-1*** at enrollment; ***visit-2*** on day-8, day-9, or day-10 of treatment cycle-1; ***visit-3*** at four weeks; ***visit-4*** at 3 months; and ***visit-5*** at 6 months. ***Visit-1*** was for collecting baseline data, obtaining biochemical blood tests, issuing a menstrual diary card, and providing the cycle-1 medication. ***Visit-2*** was for obtaining endometrial sampling. ***Visit-3*** was for evaluating compliance and side effects, and for providing cycle-2 and cycle-3 medication. ***Visit-4*** was for providing cycle-4 to cycle-6 medication. ***Visit-5*** was for follow-up biochemical blood tests.

All participants were instructed to record data relating to the following in their menstrual diary cards: day of taking trial medication, possible side effects, and vaginal bleeding. One diary card was used to record data from one treatment cycle. The participants had to return the diary card and the leftover medication at the following visit.

### Trial medication and treatment allocation

The trial medication was 10 mg MPA or 150 µg DSG administered orally once daily before bedtime for 10 consecutive days per month for 6 months. Each dose of both study medications was encapsulated in a white capsule that was identical in physical appearance. Ten capsules of the same medicines were pre-packed in a zip-locked opaque plastic bag labeled with a serial number according to the randomization code. Patients were allocated to either the control group (MPA) or the study group (DSG) by simple randomization using a random allocation software program^29^. The randomization codes were individually contained in sealed opaque envelopes sequentially numbered. An independent nurse who had no contact with study participants opened each envelope and dispensed the assigned package of trial medication. The packaging of trial medications and the generation of randomization codes were produced by an independent pharmacist of the Department of Pharmacy. Therefore, the gynecologists and nurses who contacted the patients, the patients themselves, and the gynecologic pathologist were blinded to the group assignments.

Instruction for a missed dose of trial medication included taking the missed dose as soon as the patients remembered or skipping the dose when it was almost the time of the next scheduled dose. In the latter case, the participants would complete the trial medication in > 10 days. The cycles with > 20% skipped doses were counted as non-compliance treatment cycles.

### Outcomes

The primary efficacy outcome was the “endometrial response**”**, which was evaluated from histopathology of endometrial biopsy on medication day-8, day-9, or day-10 of treatment cycle-1. Endometrial biopsy was performed using a disposable, flexible cannula with its own internal suction piston (Endocell®, Wallach Surgical Devices, Trumbull, CT, USA). The endometrial tissue was immediately fixed in formalin and sent to the Department of Pathology for routine tissue processing with hematoxylin and eosin staining. The tissue section was examined under a light microscope by a gynecologic pathologist (M.W.). Endometrial histopathology was categorized into (i)* complete pseudodecidualization* if the entire endometrial biopsy specimen showed pseudodecidual transformation, (ii)* incomplete pseudodecidualization* if the specimen showed uneven pseudodecidual transformation, (iii)* treatment failure* if the specimen showed proliferative endometrium or endometrial neoplasia, and (iv) *undetermined* if the specimen was inadequate for evaluation. The success rate of endometrial response was the proportion of the cases with complete pseudodecidualization in the intention-to-treat cases. Dropout cases were counted as treatment failure cases.

The secondary efficacy outcome was the “clinical response”, which was evaluated from characteristics of withdrawal bleeding of treatment cycle 2 to cycle 6. The withdrawal bleeding was defined as bleeding that occurred after completion of each course of trial medication and before starting a new one (i.e., between days 11 and 28 of the treatment cycle). The amount of bleeding was categorized according to the participant’s experience^30^ into (a)* light bleeding or spotting* (less than normal menstruation), (b) *normal bleeding* (like normal menstruation), and (c)* heavy bleeding* (more than normal menstruation). The bleeding pattern was categorized^1^ into (i)* normal bleeding* (bleeding lasted up to eight days), (ii) *prolonged bleeding* (bleeding lasted more than eight days), (iii) *breakthrough bleeding* (bleeding occurred on the day of taking medication), and (iv)* no bleeding* (no bleeding during the medication-free days. The success rate of clinical response was the proportion of the treatment cycles with acceptable bleeding [i.e., the cycles with bleeding characteristics (i-a), (i-b), or (iv)] in the intention-to-treat cycles. Missing data were imputed from the last observation carried forward.

*Safety of treatment* was evaluated from the changes in biochemical blood tests from baseline to month-6, and from side effects that occurred at any time during the study period. Biochemical blood tests were performed at ***visit-1*** and ***visit-5.*** A venous blood sample was drawn from an antecubital vein during the 08.00 to 10.00 time period after overnight fasting for 12 h. The blood sample was examined for fasting blood glucose and lipid profile (total cholesterol, triglycerides, HDL-C, and LDL-C). All biochemical assays were performed at the Department of Clinical Pathology (central laboratory certified by IS0 15,189). All assays were performed using automatic analyzers, and all assays had intra-assay and inter-assay coefficients of variation (CV) of < 5%.

### Sample size

The sample size was calculated from the primary outcome to determine the non-inferiority of DSG compared with MPA regarding the success rate of endometrial response. A free online sample size calculator was used for comparing two proportions: 2-sample non-inferiority or superiority (HyLown Consulting LLC, GA, USA). Given that the non-inferiority margin was − 10% (δ = − 0.1), the success rate of endometrial response derived from our pilot study in the DSG (θ1) and the MPA (θ2) groups was 80% and 60%, respectively. The required sample size with the equal allocation (r = 1) to achieve 90% power and an α of 0.025 was 47 patients per group. To compensate for 5% dropouts, the final sample size was 50 patients per group.

### Statistical analysis

Data were analyzed using SPSS Statistics software version 18.0 (SPSS, Inc., IL, USA). Statistical analyses of the efficacy outcomes were based on the intention-to-treat populations, and those of the safety outcomes were based on the per-protocol population. Data are presented as mean ± standard deviation (SD), median and interquartile range, or number and percent, as appropriate. Shapiro–Wilk test and Q-Q plot were used to examine the normality of continuous data. Unpaired *t*-test or Mann–Whitney U test was used to compare continuous data. Chi-square test or Fisher’s exact test was used to compare categorical data. Generalized estimating equations model was used to test the difference in the rate of acceptable bleeding from all treatment cycles between DSG and MPA groups. All statistical tests to determine differences between groups were 2-sided, and a *P-*value of less than 0.05 was considered statistically significant.

Non-inferiority was considered if the lower bound of the 95% confidence interval (CI) for the difference (Δ) in the favorable outcome lay above the non-inferiority limit. Therefore, DSG would be considered non-inferior to MPA if the lower bound for the difference in the endometrial response rate lay above − 10%.

### Ethical approval

The protocol for this study was approved by the Siriraj Institutional Review Board (SIRB) of the Faculty of Medicine Siriraj Hospital, Mahidol University (certificate of approval no. Si394/2013, date of approval 8 July 2013). The protocol was also registered with ClinicalTrials.gov (reg. no. NCT02103764, date of approval 18 Feb 2014). The initial participant enrollment was on 11 March 2014. A written informed consent was obtained from all participants before enrollment.

## Abbreviations

AUB-O: Abnormal uterine bleeding-ovulatory dysfunction
DSG: Desogestrel
MPA: Medroxyprogesterone acetate
